# Acute rather than insidious symptom onset is associated with better postoperative outcomes after transtibial pull‐out repair for medial meniscus posterior root tears

**DOI:** 10.1002/jeo2.70948

**Published:** 2026-10-09

**Authors:** Zheng‐Zheng Zhang, Shu‐Yang Guo, Wen‐Nan Xu, Jia‐Hao Zhu, Jia‐Long Luo, Jing‐Song Wang, Chuan Jiang, Bo He, Wei‐Ping Li

**Affiliations:** ^1^ Department of Sports Medicine, Sun Yat‐sen Memorial Hospital Sun Yat‐sen University Guangzhou China; ^2^ Department of Anesthesiology, Sun Yat‐sen Memorial Hospital Sun Yat‐sen University Guangzhou China

**Keywords:** acute onset, insidious onset, medial meniscus posterior root tear, meniscal extrusion, transtibial pull‐out repair

## Abstract

**Purpose:**

To compare clinical and radiologic outcomes after transtibial pull‐out repair for medial meniscus posterior root tears (MMPRTs) between patients with acute and insidious symptom onset.

**Methods:**

Patients who underwent transtibial pull‐out repair for MMPRTs between March 2022 and December 2023 and had a minimum 2‐year follow‐up were retrospectively reviewed. Patients were classified into an acute‐onset group, defined by abrupt symptom onset during or immediately after a clear inciting knee incident, or an insidious‐onset group, defined by gradually progressive symptoms without a definite precipitating event. Outcomes included patient‐reported outcome measures (PROMs) and radiologic parameters. PROMs comprised the International Knee Documentation Committee (IKDC) and Lysholm scores, with minimal clinically important difference (MCID) achievement also assessed. Radiologic outcomes included Kellgren–Lawrence (KL) grade progression, meniscal extrusion and magnetic resonance imaging (MRI) healing status.

**Results:**

A total of 49 patients were included (28 acute‐onset, 21 insidious‐onset). Both groups showed significant improvement in IKDC and Lysholm scores, with similar MCID achievement rates. At final follow‐up, the Acute Group had higher median IKDC scores than the Insidious Group (73.0 [66.8–79.0] vs. 67.0 [62.0–69.0]; *p* = 0.02) and Lysholm scores (76.0 [73.0–78.0] vs. 71.0 [70.0–74.0]; *p* < 0.01). KL‐grade progression was less frequent in the Acute Group (1/28 [3.6%] vs. 8/21 [38.1%]; *p* < 0.01). Meniscal extrusion was also lower (3.3 [2.9–3.5] vs. 3.9 [3.1–4.0] mm; *p* = 0.01), although the absolute difference was small. MRI healing status did not differ between groups.

**Conclusion:**

Acute‐onset MMPRTs were associated with better postoperative functional outcomes and less radiologic progression than insidious‐onset MMPRTs, although the clinical relevance of the small difference in meniscal extrusion remains uncertain. Therefore, symptom‐onset pattern may provide potentially useful information for preoperative prognostic counselling, but this association should be interpreted cautiously and confirmed in prospective studies.

**Level of Evidence:**

Level III.

**Abbreviations**
ANCOVA: analysis of covariance
CI: confidence interval
HKA: hip–knee–ankle
ICC: intraclass correlation coefficient
ICRS: International Cartilage Repair Society
IKDC: International Knee Documentation Committee
KL: Kellgren–Lawrence
MCID: minimal clinically important difference
ME: meniscal extrusion
MMA: modified Mason–Allen stitch
MMPRT: medial meniscus posterior root tear
MRI: magnetic resonance imaging
OR: odds ratio
PROM: patient‐reported outcome measure
PTS: posterior tibial slope

## INTRODUCTION

Medial meniscus posterior root tear (MMPRT), a distinct subgroup of meniscal lesions, accounts for 10%–21% of all meniscal tears and represents a pathologically unique condition increasingly recognized [1, 11, 28]. The aetiology of most MMPRT is degenerative. Although it is well known that the healing potential of degenerative meniscal tears after repair is low, most surgeons still recommend repair for MMPRT because of the associated meniscal extrusion (ME), which has been shown to be related to loss of meniscal function and accelerated osteoarthritis in the affected compartment.

Clinically, MMPRT patients exhibit heterogeneous symptomatology [4]. The onset pattern can generally be classified into two types [5]. One is acute symptom onset, in which patients report some well‐delineated incident, such as sudden posterior knee pain during deep knee flexion, sometimes accompanied by a popping sensation. The other is insidious onset, in which symptoms develop gradually without a definite injury and usually present as progressive pain, swelling or a sense of knee weakness. These differences may influence the reparability and biological healing potential of the meniscus and consequently contribute to different postoperative outcomes. The factors underlying this variability have not been sufficiently investigated.

Arthroscopic transtibial pull‐out repair is widely used for the treatment of this lesion. However, the procedure does not reliably reduce ME, and postoperative outcomes remain variable [10, 14, 21, 30]. Most previous studies on MMPRT repair have focused on surgical techniques or the overall outcomes of unselected patient cohorts [9, 25]. But, evidence on the outcomes of isolated MMPRT repair according to different symptom‐onset patterns remains limited [16]. Additionally, few studies specifically evaluate patient‐reported outcome measures (PROMs) and radiological parameters based on distinct MMPRT‐related symptom patterns [13, 19]. Therefore, identifying preoperative factors associated with clinical and radiological outcomes after MMPRT repair may improve prognostic stratification and patient counselling. Determining whether symptom onset patterns are associated with postoperative recovery may help address an important gap in the preoperative prognostic stratification of patients undergoing MMPRT repair.

The aim of this study was to compare the clinical and radiologic outcomes after transtibial pull‐out repair between patients with acute onset and insidious onset MMPRTs. We hypothesized that patients with an acute onset of symptoms after MMPRT would have better 2‐year outcomes, especially less ME on postoperative magnetic resonance imaging (MRI), compared to those with an insidious onset of symptoms.

## METHODS

### Study design and ethics

This retrospective cohort study was conducted at Sun Yat‐sen Memorial Hospital, Sun Yat‐sen University. The study protocol was approved by the Ethics Committee of Sun Yat‐sen Memorial Hospital, Sun Yat‐sen University (approval No. SYSEC. 2‐2024‐BA‐615). Informed consent for the use of clinical and imaging data for research purposes was obtained from all participants.

### Inclusion and exclusion criteria

Medical records of MMPRTs (Type 2 in LaPrade classification) patients who underwent transtibial pull‐out suture repair by one senior surgeon at a single institution (March 2022–December 2023) were retrospectively reviewed. Patients were excluded if: (1) follow‐up <2 years, (2) concomitant osteotomy, (3) concomitant ligament surgery, (4) concomitant centralization surgery, (5) Kellgren–Lawrence (KL) Grade ≥3 osteoarthritis, (6) affected knee subchondral insufficiency fracture or (7) incomplete radiographic (MRI or X‐ray) or clinical data.

### Identification and classification of MMPRTs

MMPRTs were initially identified on preoperative MRI and subsequently confirmed by diagnostic arthroscopy. The final tear subtype was determined intraoperatively according to the morphology‐based classification system described by LaPrade et al. [21]. Type 2 MMPRT was defined as a complete radial tear located within 9 mm of the bony attachment of the medial meniscus posterior root. Tear morphology was determined by the operating surgeon based on the arthroscopic findings and was retrospectively verified using the operative records and available arthroscopic images. Only patients with Type 2 MMPRTs were included in the present study.

### Classification of symptom onset pattern

Patients were classified into the acute symptom‐onset group or the insidious‐onset group according to the documented pattern of symptom onset rather than the presumed traumatic or degenerative nature of the meniscal tear. Acute symptom onset was defined as an abrupt onset of knee symptoms during or immediately after a clearly identifiable low‐energy knee‐loading incident, such as deep knee flexion, squatting or twisting. These incidents were not considered true traumatic injuries but were regarded as events that may have precipitated structural failure in meniscal tissue with underlying degenerative changes. Insidious onset was defined as the gradual development of knee symptoms without a clearly identifiable precipitating incident.

The onset pattern was independently classified by two orthopaedic surgeons based on the patients' medical records and the history documented at the initial clinical evaluation. Cases with uncertain or inconsistent histories were reviewed jointly, and the final classification was determined by consensus.

### Surgical indication

In our institution, symptomatic patients with repairable MMPRTs, regardless of whether symptom onset was acute or insidious, who met the predefined surgical criteria were considered directly for surgical repair rather than routinely undergoing an initial trial of non‐operative treatment. This approach was based on the concern that persistent disruption of the medial meniscus posterior root may compromise hoop function and contribute to continued ME and degenerative progression. The decision for surgery was made after consideration of patient age, radiographic osteoarthritis, coronal alignment, tissue reparability and willingness to adhere to postoperative rehabilitation. Specifically, our institution's transtibial pull‐out suture repair indications for MMPRT required patients to meet all criteria: ≤70 years old; KL Grade ≤2 radiographic osteoarthritis; absence of pathological varus alignment, defined as a mechanical hip–knee–ankle (HKA) angle ≤10° on full‐length weight‐bearing anteroposterior radiographs of the lower extremities; and willingness to adhere to strict postoperative rehabilitation.

### Surgical technique and post‐operative rehabilitation

After general anaesthesia induction, diagnostic arthroscopy was performed via standard portals through standard anterolateral and anteromedial portals using a 30° arthroscope to confirm MMPRT with adequate tissue quality for repair. The posteromedial compartment was not entered, and a 70° arthroscope was not used. Percutaneous pie‐crusting of the medial collateral ligament was performed in all patients under valgus stress to enlarge the medial joint space and improve visualization of and access to the medial meniscus posterior root.

Articular cartilage lesions were evaluated intraoperatively according to the International Cartilage Repair Society (ICRS) classification [6]. Limited mechanical chondroplasty was performed for ICRS Grade 1–2 lesions when fibrillated or unstable cartilage tissue was identified. The procedure consisted of mechanical debridement of unstable cartilage tissue to create a stable cartilage rim. No additional cartilage repair procedures, such as microfracture or multiple drilling, were performed because no isolated full‐thickness cartilage lesions were identified in the current cohort.

For repair, the anatomic medial meniscus posterior root footprint was prepared using curettes and a shaver, followed by creation of 1–2 transtibial tunnels from the anteromedial tibial cortex to the footprint. Two nonabsorbable sutures (cinch or modified Mason‐Allen stitch) were passed through the posterior medial meniscus and retrieved through the tunnels.

The sutures were then passed through a suture anchor or cortical button, appropriately tensioned and fixed on the anterior tibia to complete the repair. Representative arthroscopic images showed the MMPRT before repair (Figure 1a) and the repaired posterior root after transtibial pull‐out fixation (Figure 1b).

**Figure 1:**
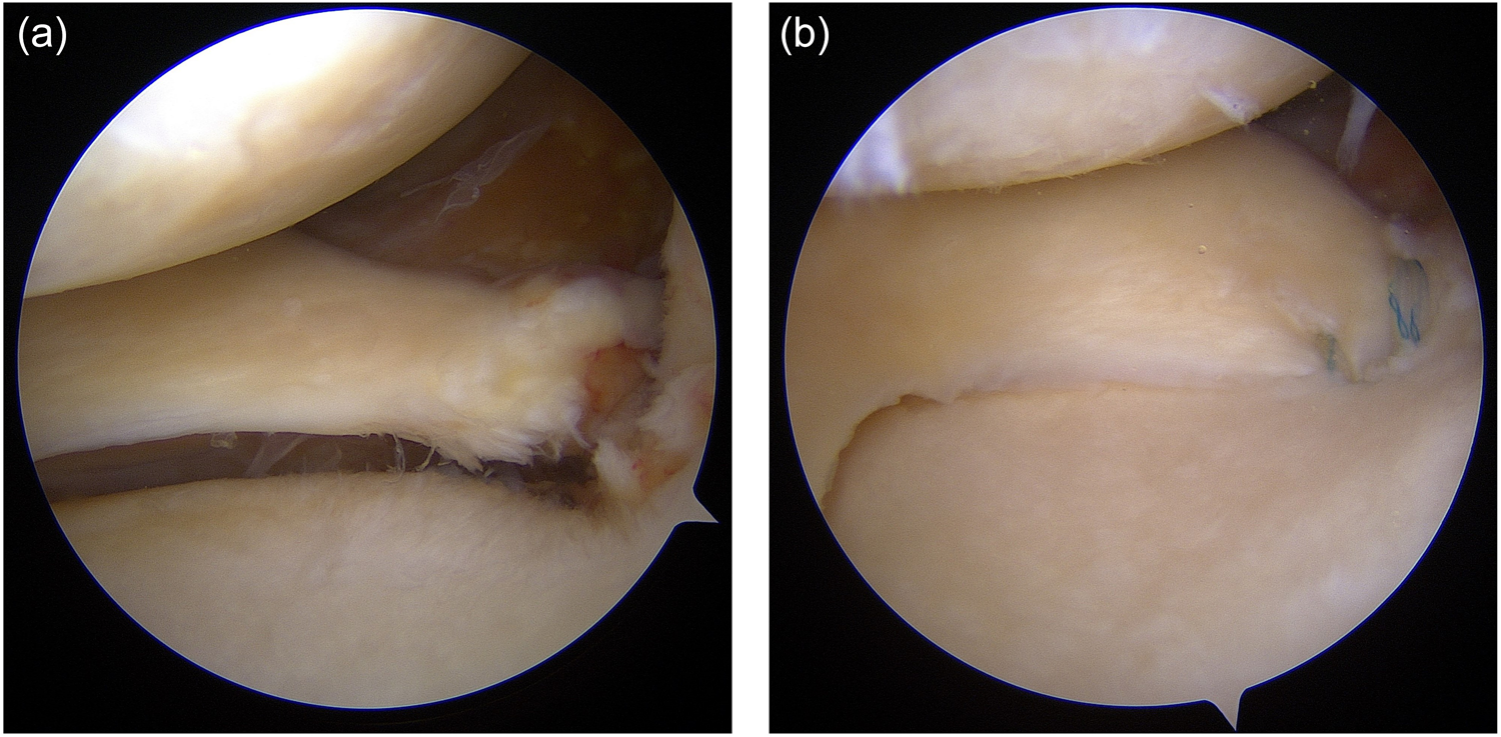
Representative intraoperative arthroscopic images of medial meniscus posterior root tear during transtibial pull‐out repair. (a) Arthroscopic views showing the medial meniscus posterior root tear before repair and (b) reduction and stabilization of the repaired posterior root after transtibial pull‐out fixation.

All patients received the same postoperative rehabilitation protocol. Affected limb non‐weightbearing was maintained for the first 6 weeks, followed by gradual weightbearing progression to full tolerance. Knee motion was restricted to 90° with a brace for 4 weeks, then range‐of‐motion exercises were extended by 10° every other week; flexion was limited to 120° within the first 3 months. All patients underwent specialized physical therapist‐led rehabilitation for ≥3 months, focusing on quadriceps strengthening.

## PATIENT EVALUATION

### Demographic and perioperative data

Demographic and perioperative data were retrospectively collected from the medical records. The recorded demographic variables included age, sex, affected side, body mass index, preoperative symptom duration and follow‐up duration. Intraoperative variables included the number of transtibial tunnels, suture configuration, fixation method and whether limited chondroplasty was performed.

### Clinical outcomes

Preoperative and final follow‐up PROMs included International Knee Documentation Committee (IKDC) subjective and Lysholm scores [26]. The IKDC subjective score at final follow‐up was designated as the sole primary outcome. The minimal clinically important difference (MCID) was used to assess clinically meaningful postoperative improvement for each score [22, 27].

### Radiographic findings

Radiographic assessment included the preoperative HKA angle, posterior tibial slope (PTS) and KL grade. Preoperative radiographs were obtained before surgery, and postoperative standing anteroposterior knee radiographs were obtained at approximately 1 year after surgery, within an assessment window of 12 ± 1 months for all patients. The HKA angle was evaluated on full‐length weight‐bearing anteroposterior radiographs of the lower extremities. The KL grade was assessed on standing anteroposterior knee radiographs preoperatively and at the postoperative 1‐year assessment. For statistical analysis, KL Grades 0 and 1 were combined into a single category. Radiographic progression was defined as progression from KL Grade 0–1 to KL Grade ≥2 or an increase from baseline KL Grade 2 to Grade ≥3; a change from KL Grade 0 to Grade 1 was not considered progression.

### MRI findings

MRI assessment included ME and the healing status of the repaired MMPRT. ME and healing status were assessed using preoperative MRI and postoperative MRI obtained at approximately 1 year after surgery, within an assessment window of 12 ± 1 months for all patients. ME was measured on coronal MRI scans according to the method described by Costa et al. [12]. Measurements were performed using the electronic caliper of the GE Healthcare picture archiving and communication system workstation and were displayed and recorded to the nearest 0.1 mm. The healing status of the repaired MMPRT was assessed on coronal, sagittal and axial MRI scans according to the method described by Kim et al., based on continuity between the repaired root and the bone attachment site [18].

All radiological and MRI assessments were independently performed by 2 experienced orthopaedic surgeons who were blinded to the patients' clinical outcomes and symptom‐onset group, using a GE Healthcare picture archiving and communication system workstation. Observer 1 performed all assessments for the entire cohort twice at a 1‐month interval to evaluate intraobserver reliability, whereas Observer 2 independently performed the assessments once. The first and second assessments of Observer 1 were compared to evaluate intraobserver reliability, and the first assessment of Observer 1 was compared with that of Observer 2 to evaluate interobserver reliability.

Single‐measure, absolute‐agreement intraclass correlation coefficients (ICCs) with 95% confidence intervals (CIs) were calculated for continuous radiological measurements, including the HKA angle, PTS and preoperative and postoperative ME. ICC values were interpreted as poor (<0.50), moderate (0.50–0.75), good (0.75–0.90) or excellent (>0.90). Quadratic weighted kappa coefficients with 95% CIs were calculated for ordinal assessments, including preoperative and postoperative KL grades. Weighted kappa values were interpreted as poor (<0.00), slight (0.00–0.20), fair (0.21–0.40), moderate (0.41–0.60), substantial (0.61–0.80) or almost perfect (0.81–1.00).

### Statistical analyses

Statistical analyses were performed using IBM SPSS Statistics for Windows, version 25.0 (IBM Corp.). Continuous variables were compared between the Acute and Insidious Groups using the two‐sided Mann–Whitney *U* test. Categorical variables were compared using the Pearson *χ* ^2^ test or Fisher's exact test. Within‐group comparisons between preoperative and final follow‐up PROMs were performed using the Wilcoxon signed‐rank test. For postoperative ME and ΔME, between‐group mean differences (Insidious minus Acute) with 95% CIs and Cohen's *d* were calculated; *d* values of 0.2, 0.5 and 0.8 indicated small, medium and large effects, respectively.

Multivariable linear regression was performed for the final IKDC and Lysholm scores, adjusting for the corresponding preoperative score, age, symptom duration, baseline KL‐grade category, baseline ME, chondroplasty, HKA angle and PTS. Postoperative 1‐year ME was analysed using an analysis of covariance (ANCOVA)‐type linear regression model adjusted for the available baseline and surgical covariates. Because only nine patients demonstrated KL‐grade progression, Firth penalized logistic regression was used with a parsimonious set of covariates. Adjusted regression coefficients and odds ratios (ORs) were reported with 95% CIs.

MCID was determined using a distribution‐based method, defined as 50% of the standard deviation of the change in outcome scores from preoperative to final follow‐up assessment. The IKDC subjective score at final follow‐up was designated as the sole primary outcome, whereas all other clinical and radiological variables were considered secondary outcomes. All statistical tests were two‐sided, and a *p* value < 0.05 was considered statistically significant.

### Sample size analysis

As this was a retrospective cohort study, no formal a priori sample size calculation was performed. The sample size was determined by the number of consecutive eligible patients treated during the predefined study period, and all patients who met the eligibility criteria were included.

## RESULTS

A total of 82 patients were screened, of whom 49 met the inclusion criteria (Figure 2). All included tears were arthroscopically classified as Type 2 MMPRTs according to the LaPrade classification. Of the 49 included patients, 28 were assigned to the Acute Group and 21 to the Insidious Group. There were no significant differences between the two groups in baseline demographic characteristics, HKA angle, PTS or intraoperative variables (Table 1). Percutaneous pie‐crusting of the medial collateral ligament was performed in all 49 patients. Limited mechanical chondroplasty for ICRS Grade 1–2 cartilage lesions was performed in eight patients, including three patients in the Acute Symptom‐Onset Group and five patients in the Insidious‐Onset Group. No full‐thickness cartilage lesions were identified in either group.

**Figure 2:**
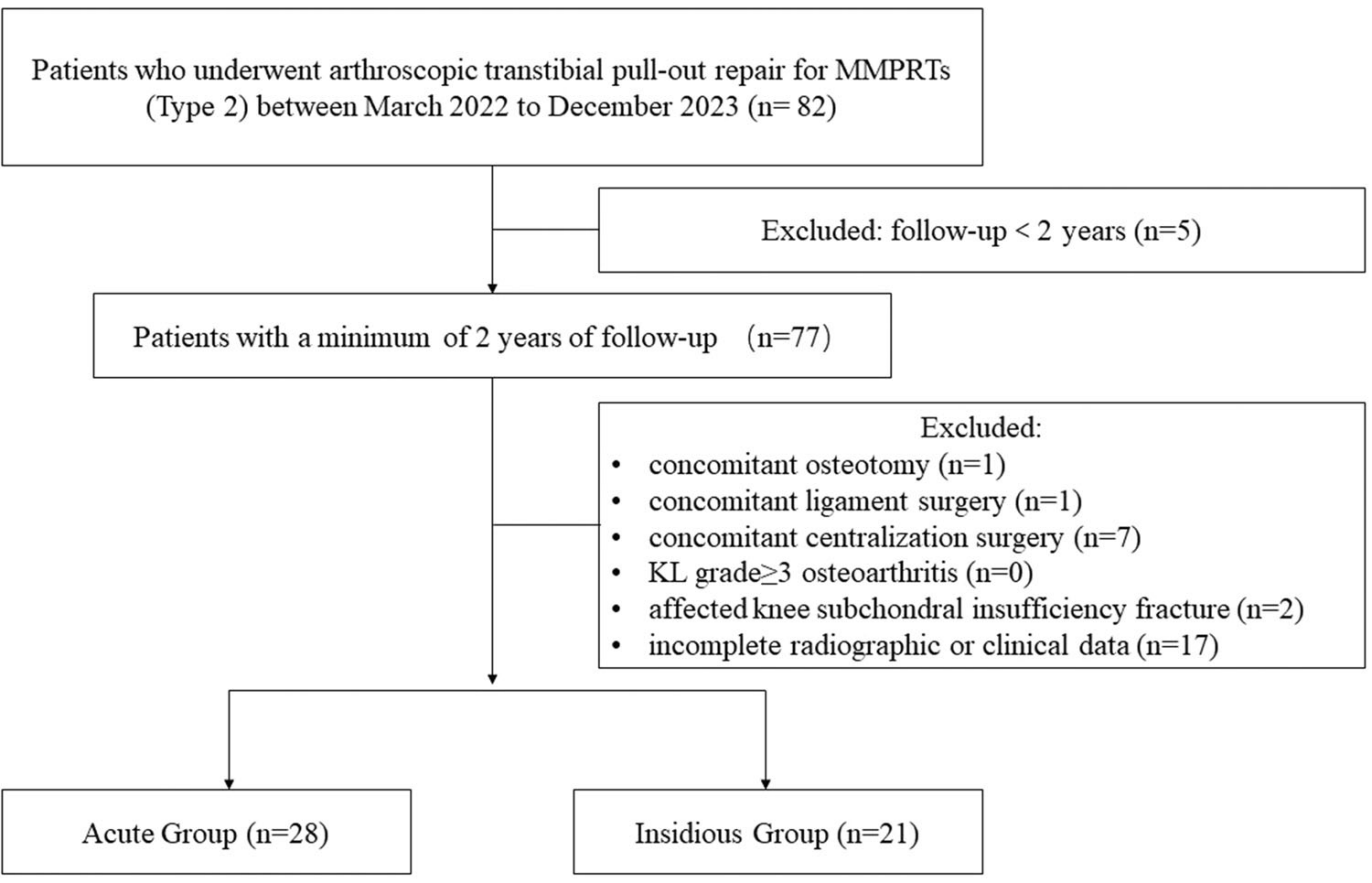
Flowchart of patient inclusion in the study. KL, Kellgren–Lawrence; MMPRT, medial meniscus posterior root tear.

**Table 1:** Baseline demographic and intraoperative characteristics.

Variables	Acute group (*n* = 28)	Insidious group (*n* = 21)	*p* value
Demographic data			
Age, years	57.5 (52.5–60.0)	56.0 (52.0–62.0)	*p* = 0.57
Sex, male/female	8 (28.6%)/20 (71.4%)	5 (23.8%)/16 (76.2%)	*p* = 0.76
Affected side, left/right	16 (57.1%)/12 (42.9%)	13 (61.9%)/8 (38.1%)	*p* = 0.78
Body mass index, kg/m^2^	24.7 (23.2–26.2)	24.8 (23.1–26.0)	*p* = 0.82
Follow‐up duration, months	24.0 (24.0–24.0)	24.0 (24.0–24.0)	*p* = 0.26
Preoperative symptom duration, months	3.0 (2.0–5.0)	3.0 (2.0–4.0)	*p* = 0.86
Radiographic parameters			
HKA angle	2.9 (2.3–3.6)	2.7 (2.1–3.7)	*p* = 0.90
PTS angle	6.5 (5.6–7.6)	6.3 (5.2–7.1)	*p* = 0.56
Intraoperative variables			
Number of tunnels, 1/2	24 (85.7%)/4 (14.3%)	18 (85.7%)/3 (14.3%)	*p* = 1.00
Suture configuration, cinch/MMA	24 (85.7%)/4 (14.3%)	18 (85.7%)/3 (14.3%)	*p* = 1.00
Fixation, anchor/button	24 (85.7%)/4 (14.3%)	18 (85.7%)/3 (14.3%)	*p* = 1.00
Chondroplasty	3 (10.7%)/25 (89.3%)	5 (23.8%)/16 (76.2%)	*p* = 0.26

*Note*: Continuous data are presented as median (interquartile range) and categorical data as *n* (%). Continuous variables were compared using the two‐sided Mann–Whitney *U* test; categorical variables were compared using the Pearson *χ* ^2^ test or Fisher exact test, as appropriate.

Abbreviations: HKA, hip–knee–ankle; MMA, modified Mason–Allen stitch; PTS, posterior tibial slope.

Both groups showed significant improvement in IKDC subjective and Lysholm scores at final follow‐up compared with the preoperative assessment (Table 2). At final follow‐up, the Acute Group had higher IKDC (73.0 [66.8–79.0] vs. 67.0 [62.0–69.0]; *p* = 0.02) and Lysholm scores (76.0 [73.0–78.0] vs. 71.0 [70.0–74.0]; *p* < 0.01) than the Insidious Group. The improvement in IKDC score was also greater in the Acute Symptom‐Onset Group (34.0 [27.8–45.0] vs. 24.0 [16.0–30.0]; *p* = 0.01), whereas the change in Lysholm score did not differ significantly between the groups (26.0 [14.0–33.0] vs. 17.0 [14.0–22.0]; *p* = 0.05). MCID achievement rates were similar between the groups for both IKDC (96.4% vs. 95.2%; *p* = 1.00) and Lysholm scores (92.9% vs. 95.2%; *p* = 1.00).

**Table 2:** Patient‐reported outcomes after transtibial pull‐out repair.

Variables	Acute group	Insidious group	*p* value
IKDC			
Preoperative	37.0 (33.0–40.3)	42.0 (36.0–45.0)	*p* = 0.10
Postoperative	73.0 (66.8–79.0)*	67.0 (62.0–69.0)*	***p*** = **0.02**
ΔIKDC	34.0 (27.8–45.0)	24.0 (16.0–30.0)	***p*** = **0.01**
MCID Achievement, yes/no	27 (96.4%)/1 (3.6%)	20 (95.2%)/1 (4.8%)	*p* = 1.00
Lysholm score			
Preoperative	49.0 (44.0–57.8)	54.0 (53.0–57.0)	*p* = 0.11
Postoperative	76.0 (73.0–78.0)*	71.0 (70.0–74.0)*	***p*** < **0.01**
ΔLysholm score	26.0 (14.0–33.0)	17.0 (14.0–22.0)	*p* = 0.05
MCID Achievement, yes/no	26 (92.9%)/2 (7.1%)	20 (95.2%)/1 (4.8%)	*p* = 1.00

*Note*: Continuous data are presented as median (interquartile range) and categorical data as *n* (%). Δ indicates the individual change from the preoperative assessment to final follow‐up. Between‐group comparisons were performed using the two‐sided Mann–Whitney *U* test. Within‐group comparisons were performed using the Wilcoxon signed‐rank test. Bold values indicate statistical significance (*p* < 0.05).

Abbreviations: IKDC, International Knee Documentation Committee; MCID, minimal clinically important difference.

* *p* < 0.05 compared with the preoperative value within the same group.

Radiographic and MRI findings are summarized in Table 3. Preoperative KL grade distribution and ME were comparable between the groups. The postoperative KL‐grade distribution differed significantly between the groups (*p* = 0.01), and KL‐grade progression was more frequent in the Insidious Group than in the Acute Group (8/21 [38.1%] vs. 1/28 [3.6%]; *p* < 0.01). Postoperative ME was also greater in the Insidious Group (3.9 [3.1–4.0] vs. 3.3 [2.9–3.5] mm; *p* = 0.01). Likewise, the increase in ME from baseline to postoperative time point was greater in the Insidious Group (0.7 [0.5–1.0] vs. 0.4 [0.1–0.8] mm; *p* = 0.01). Postoperative healing status on MRI did not differ significantly between the two groups (*p* = 0.47).

**Table 3:** Radiologic outcomes after transtibial pull‐out repair.

Variables	Acute group	Insidious group	*p* value
KL grade			
Preoperative, 0–1^a^/2/3	26 (92.9%)/2 (7.1%)/0	20 (95.2%)/1 (4.8%)/0	*p* = 1.00
Postoperative, 0–1/2/3	26 (92.9%)/1 (3.6%)/1 (3.6%)	13 (61.9%)/7 (33.3%)/1 (4.8%)	***p*** = **0.01**
Progression of KL grade, yes/no	1 (3.6%)/27 (96.4%)	8 (38.1%)/13 (61.9%)	***p*** < **0.01**
ME, mm			
Preoperative	2.8 (2.4–3.2)	3.2 (2.4–3.3)	*p* = 0.44
Postoperative	3.3 (2.9–3.5)	3.9 (3.1–4.0)	***p*** = **0.01**
Δ ME, mm	0.4 (0.1–0.8)	0.7 (0.5–1.0)	***p*** = **0.01**
Healing of MMPRT, complete/partial/no	24 (85.7%)/4 (14.3%)/0	16 (76.2%)/5 (23.8%)/0	*p* = 0.47

*Note*: Continuous data are presented as median (interquartile range) and categorical data as *n* (%). ΔME was calculated as postoperative 1‐year ME minus preoperative ME. A positive value indicates an increase in ME after surgery. Additional effect estimates were calculated as the insidious‐onset group minus the acute symptom onset group. For postoperative ME, the mean difference was 0.4 mm (95% CI, 0.1–0.7 mm; Cohen's *d* = 0.76). For ΔME, the mean difference was 0.3 mm (95% CI, 0.1–0.6 mm; Cohen's *d* = 0.65). Bold values indicate statistical significance (*p* < 0.05).

Abbreviations: CI, confidence interval; KL, Kellgren–Lawrence; ME, meniscal extrusion; MMPRT, medial meniscus posterior root tear; MRI, magnetic resonance imaging.

a KL Grades 0 and 1 were combined into a single category for statistical analysis. Progression from KL Grade 0 to Grade 1 was not considered radiographic progression.

Reliability analyses demonstrated excellent intraobserver reliability and good‐to‐excellent interobserver reliability for all continuous radiological measurements. The intraobserver ICCs ranged from 0.989 to 0.998, whereas the interobserver ICCs ranged from 0.849 to 0.974. Quadratic weighted kappa coefficients demonstrated almost perfect intraobserver and interobserver agreement for both preoperative and postoperative KL grading. Detailed reliability estimates and 95% CIs are presented in Table 4.

**Table 4:** Intraobserver and interobserver reliability of radiological assessments.

Radiological assessment	Statistic	Intraobserver estimate (95% CI)	Interobserver estimate (95% CI)
HKA angle	ICC	0.996 (0.993–0.998)	0.949 (0.920–0.966)
Posterior tibial slope	ICC	0.998 (0.996–0.999)	0.974 (0.959–0.983)
Preoperative meniscal extrusion	ICC	0.989 (0.981–0.993)	0.877 (0.790–0.921)
Postoperative meniscal extrusion	ICC	0.990 (0.982–0.994)	0.849 (0.758–0.900)
Preoperative KL grade	Weighted *κ*	0.856 (0.715–0.965)	0.843 (0.720–0.941)
Postoperative KL grade	Weighted *κ*	0.895 (0.787–0.964)	0.900 (0.773–0.978)

*Note*: ICCs were calculated using single‐measure, absolute‐agreement models for continuous variables. Quadratic weighted kappa coefficients were calculated for ordinal KL grading. Intraobserver reliability was evaluated by comparing the first and second assessments of Observer 1. Interobserver reliability was evaluated by comparing the first assessment of Observer 1 with the assessment of Observer 2.

Abbreviations: CI, confidence interval; HKA, hip–knee–ankle; ICC, intraclass correlation coefficient; KL, Kellgren–Lawrence.

Adjusted analyses are summarized in Table 5. After adjustment for relevant baseline and surgical variables, acute symptom onset remained associated with higher final IKDC scores (adjusted *β* = 6.8; 95% CI, 1.5–12.2; *p* = 0.01), higher final Lysholm scores (adjusted *β* = 2.5; 95% CI, 0.4–4.6; *p* = 0.02) and lower postoperative 1‐year ME (adjusted *β* = −0.32 mm; 95% CI, −0.55 to −0.08; *p* = 0.01). Acute symptom onset was also associated with lower odds of KL‐grade progression in the Firth penalized logistic regression model (adjusted OR = 0.02; 95% CI, 0.001–0.35; *p* < 0.01). Given the small number of KL‐grade progression events (*n* = 9), the adjusted OR should be interpreted cautiously because of the potential instability of the model estimate. Although the adjusted difference in postoperative ME was statistically significant, the absolute difference was small.

**Table 5:** Adjusted associations between symptom‐onset pattern and postoperative outcomes.

Outcome	Adjusted effect	95% CI	*p* value
Final IKDC score	*β* = 6.8 points	1.5–12.2	***p*** = **0.01**
Final Lysholm score	*β* = 2.5 points	0.4–4.6	***p*** = **0.02**
Postoperative ME	*β* = −0.32 mm	−0.55 to −0.08	***p*** = **0.01**
KL grade progression	OR = 0.02	0.001–0.35	***p*** < **0.01**

*Note*: Adjusted effects compare the Acute Group with the Insidious Group as the reference. Positive *β* values indicate higher final scores in the Acute Group, whereas a negative *β* value for ME indicates less postoperative extrusion. An OR below 1 indicates lower odds of KL‐grade progression. Bold values indicate statistical significance (*p* < 0.05).

Abbreviations: *β*, regression coefficient; CI, confidence interval; IKDC, International Knee Documentation Committee; KL, Kellgren–Lawrence; ME, meniscal extrusion; OR, odds ratio.

## DISCUSSION

The most important finding of the present study was that acute symptom onset was associated with better postoperative clinical and radiologic outcomes than insidious onset after transtibial pull‐out repair for MMPRTs. Although both groups demonstrated significant improvements in IKDC and Lysholm scores, the Acute Symptom‐Onset Group had higher final PROMs, less postoperative ME, and a lower frequency of KL‐grade progression than the Insidious‐Onset Group, whereas postoperative healing status on MRI was comparable between groups. After adjustment for relevant baseline and surgical variables, acute symptom onset remained associated with higher final IKDC and Lysholm scores, lower postoperative 1‐year ME and lower odds of KL‐grade progression. These findings indicate an association rather than a causal relationship and suggest that symptom onset pattern may be associated with prognosis after MMPRT repair.

The radiological findings also suggest that apparent structural healing does not necessarily indicate equivalent restoration of meniscal position or biomechanical function. Postoperative MRI healing status was similar between groups, whereas the Insidious‐Onset Group showed greater ME and KL‐grade progression. This suggests that MRI‐assessed healing mainly reflects continuity of the repaired root and may not fully represent restoration of overall meniscal position or protection against joint degeneration [3, 20, 26]. The greater progression of ME in the Insidious Group may indicate a less favourable postoperative biomechanical environment and may further promote degenerative changes in the joint [17, 24, 25, 29]. These structural changes are commonly associated with knee pain and functional limitation and may partly explain the lower final PROM scores in this group. However, the absolute between‐group differences in ME were small (0.3–0.4 mm), and their clinical relevance remains uncertain because no validated clinically meaningful threshold is available.

A possible explanation for these findings is that the two onset patterns reflect different disease states at the time of surgery. Acute symptom onset may reflect an acutely recognized structural failure in meniscal tissue with relatively less advanced degeneration, whereas insidious onset may more often reflect chronic overload and degenerative change. This difference may also be related to surgical timing. A recent systematic review showed that MMPRT repair led to significant improvement even when surgery was delayed, but earlier repair was associated with greater gains in patient‐reported outcomes than delayed repair. The authors concluded that delayed repair can still provide worthwhile improvement, but early treatment should be pursued whenever possible [23]. This is particularly relevant because patients with acute MMPRT are usually identified earlier because of a clear inciting event, whereas patients with insidious MMPRT often present later because of the absence of a definite cause and the subtle nature of symptoms. Therefore, the less favourable outcomes in the Insidious Group may be related to a longer period of abnormal joint loading and a more degenerative tissue environment before surgery. These unfavourable baseline conditions may not be fully reversed by surgical repair alone.

From a clinical perspective, onset pattern is simple to identify before surgery and may therefore have practical value as a preoperative prognostic factor [5, 16, 23, 25]. This is particularly relevant because improving preoperative risk stratification remains an important issue in MMPRT repair, where postoperative outcomes are heterogeneous despite technically successful restoration of root continuity. However, it should not be used as an isolated indication for or against repair. To facilitate the clinical interpretation of PROMs, we used MCID. MCID mainly reflects whether a patient has achieved a minimally meaningful improvement. This threshold allows a more objective interpretation of subjective PROMs [2, 7, 8, 15]. MCID achievement rates were similar between the groups, suggesting that both acute‐onset and insidious‐onset patients generally benefited from surgery. Surgical decision‐making should therefore continue to incorporate age, symptom duration, baseline KL grade, ME, cartilage status, coronal alignment, PTS and the patient's ability to comply with postoperative rehabilitation. Larger prospective studies are required to determine whether symptom‐onset pattern adds prognostic value beyond these established clinical and radiological factors.

This study has several limitations. First, the retrospective design precludes causal inference and may have introduced selection bias, recall bias and misclassification of symptom‐onset pattern. Because symptom‐onset pattern was determined retrospectively from medical records and the documented clinical history rather than from a standardized prospective assessment, misclassification of symptom onset cannot be completely excluded. Second, the relatively small sample size, absence of an a priori sample size calculation, and limited number of KL‐grade progression events restricted the adjusted analyses. Although Firth penalized logistic regression was used to reduce small‐sample bias, the model estimates, particularly the adjusted OR for KL‐grade progression, may remain unstable and should therefore be interpreted cautiously. Third, clinical follow‐up was limited to a minimum of 24 months, whereas radiographic and MRI outcomes were assessed at approximately 1 year, limiting evaluation of longer‐term clinical and structural progression. Finally, some procedural variation remained despite exclusion of centralization cases, and the small between‐group differences in ME may have been affected by manual MRI measurement variability. The single‐surgeon, single‐institution cohort and strict surgical indications may also limit generalizability.

In summary, both acute symptom‐onset and insidious‐onset MMPRTs showed meaningful clinical improvement after transtibial pull‐out repair. Acute symptom onset was associated with higher postoperative IKDC and Lysholm scores and a lower risk of radiographic KL‐grade progression than insidious onset. Although acute symptom onset was also associated with less postoperative ME, the absolute difference was small and its clinical relevance remains uncertain. Symptom‐onset pattern may therefore provide potentially useful information for preoperative prognostic stratification, but its prognostic value requires confirmation in larger prospective studies. Given the retrospective design, small sample size, possible recall bias and potential residual confounding, these findings should be interpreted cautiously.

## AUTHOR CONTRIBUTIONS

*Conceptualization*: Zheng‐Zheng Zhang and Wen‐Nan Xu. *Methodology*: Zheng‐Zheng Zhang, Wen‐Nan Xu, Jing‐Song Wang, Chuan Jiang, Wei‐Ping Li and Bo He. *Data curation*: Zheng‐Zheng Zhang, Wen‐Nan Xu and Bo He. *Formal analysis*: Zheng‐Zheng Zhang and Shu‐Yang Guo. *Investigation*: Zheng‐Zheng Zhang, Jia‐Hao Zhu and Jia‐Long Luo. *Writing—original draft preparation*: Zheng‐Zheng Zhang, Shu‐Yang Guo and Jia‐Hao Zhu. *Writing—review and editing*: All authors. *Supervision*: Zheng‐Zheng Zhang, Wen‐Nan Xu, Jing‐Song Wang, Chuan Jiang, Wei‐Ping Li and Bo He. *Project administration*: Zheng‐Zheng Zhang and Bo He. All authors have read and approved the final manuscript.

## CONFLICT OF INTEREST STATEMENT

The authors declare no conflicts of interest.

## ETHICS STATEMENT

This study was approved by the Ethics Committee of Sun Yat‐sen Memorial Hospital, Sun Yat‐sen University (approval no. SYSEC. 2‐2024‐BA‐615). The study was conducted in accordance with the Declaration of Helsinki and relevant ethical requirements. Informed consent for the use of clinical and imaging data for research purposes was obtained from all participants.

## Data Availability

The data supporting the findings of this study are available from the corresponding authors upon reasonable request.

## References

[1] Allende F , García JR , Ayala SG , Dzidzishvili L , Quiroga G , Allahabadi S , et al. Medial meniscal posterior root tears are associated with steeper medial posterior tibial slope and varus alignment. Arthrosc Sports Med Rehabilitation. 2025;7(1):100998. 10.1016/j.asmr.2024.100998 PMC11873484 40041827

[2] Allende F , Saad Berreta R , Khan ZA , Ayala SG , Carpenter ML , Rafael Garcia J , et al. Establishing the minimal clinically important difference and patient acceptable symptom state after isolated arthroscopic posterior medial meniscal root repair. Arthrosc Sports Med Rehabil. 2025;13(7):23259671251326940. 10.1177/23259671251326940 PMC12246519 40656651

[3] Aşci M , Şahbat Y , Gedikbaş M , Sobay U , Erpala F , Güneş T . Arthroscopic all‐suture anchor repair of medial meniscus posterior root tears without a posteromedial portal: clinical improvement and healing despite persistent extrusion. J Clin Med. 2025;14(23):8272. 10.3390/jcm14238272 PMC12692641 41375575

[4] Banovetz MT , Roethke LC , Rodriguez AN , LaPrade RF . Meniscal root tears: a decade of research on their relevant anatomy, biomechanics, diagnosis, and treatment. Arch Bone Jt Surg. 2022;10(5):366–380. 10.22038/ABJS.2021.60054.2958 PMC9194705 35755791

[5] Berreta RS , Pallone L , Mameri ES , Gonzalez F , Rubin J , Manivannan A , et al. Traumatic onset of medial meniscus posterior root tears is associated with younger age while insidious onset is associated with chronic overload and bone bruising. Arthrosc Sports Med Rehabil. 2025;7(3):101118. 10.1016/j.asmr.2025.101118 PMC12276536 40692919

[6] Brittberg M , Winalski CS . Evaluation of cartilage injuries and repair. J Bone Jt Surg Am. 2003;85(suppl_2):58–69. 10.2106/00004623-200300002-00008 12721346

[7] Byun J , Kim SH , Choi CH , Jung M , Chung K , Jung SH , et al. Patients with preoperative bone marrow oedema benefit more substantially from medial meniscus posterior root repair. Knee Surg Sports Traumatol Arthrosc. 2026;34(3):834–843. 10.1002/ksa.12792 PMC12948345 40679252

[8] Chahla J , Saad Berreta R , Cotter EJ , Villarreal‐Espinosa JB , Boden S , McCormick J , et al. Minimum 5‐year outcomes, complication rates, and conversion to total knee arthroplasty following isolated medial meniscus posterior root repair. Orthop J Sports Med. 2025;13(11):23259671251365977. 10.1177/23259671251365977 PMC12612512 41244229

[9] Chang PS , Radtke L , Ward P , Brophy RH . Midterm outcomes of posterior medial meniscus root tear repair: a systematic review. Am J Sports Med. 2022;50(2):545–553. 10.1177/0363546521998297 33780278

[10] Chung KS , Ha JK , Ra HJ , Nam GW , Kim JG . Pullout fixation of posterior medial meniscus root tears: correlation between meniscus extrusion and midterm clinical results. Am J Sports Med. 2017;45(1):42–49. 10.1177/0363546516662445 27558809

[11] Cinque ME , Chahla J , Moatshe G , Faucett SC , Krych AJ , LaPrade RF . Meniscal root tears: a silent epidemic. Br J Sports Med. 2018;52(13):872–876. 10.1136/bjsports-2017-098942 29574455

[12] Costa CR , Morrison WB , Carrino JA . Medial meniscus extrusion on knee MRI: is extent associated with severity of degeneration or type of tear? Am J Roentgenol. 2004;183(1):17–23. 10.2214/ajr.183.1.1830017 15208101

[13] Elnewishy A , Elsenosy AM , Nahas S , Abdalla M , Symeon N , Teama H , et al. A systematic review and meta‐analysis of medial meniscus root tears: Is surgery the key to better outcomes? Cureus. 2024;16(12):e75199. 10.7759/cureus.75199 PMC11700375 39759717

[14] Feucht MJ , Kühle J , Bode G , Mehl J , Schmal H , Südkamp NP , et al. Arthroscopic transtibial pullout repair for posterior medial meniscus root tears: a systematic review of clinical, radiographic, and second‐look arthroscopic results. Arthroscopy. 2015;31(9):1808–1816. 10.1016/j.arthro.2015.03.022 25979687

[15] Garcia JR , Allende F , Atkins MA , McCormick JR , Yanke AB , Cole BJ , et al. Patient‐reported outcomes measurement information system captures clinically meaningful improvement after transtibial pull‐out repair of medial meniscal posterior root tears: two‐year outcomes. Arthroscopy. 2025;41:4180–4190. 10.1016/j.arthro.2025.04.014 40268070

[16] Gerhold C , Carpenter M , Khan BA ZA , Bernardo Villarreal‐Espinosa J , Verma N , Chahla J . Paper 19: medial meniscus posterior root tears with an insidious onset have poorer outcomes following transtibial pull‐out repair. Orthop J Sports Med. 2025;13(9_suppl3):2325967125S00076.

[17] Kawada K , Furumatsu T , Yokoyama Y , Higashihara N , Tamura M , Ozaki T . Meniscal healing status after medial meniscus posterior root repair negatively correlates with a midterm increase in medial meniscus extrusion. Knee Surg Sports Traumatol Arthrosc. 2024;32(9):2219–2227. 10.1002/ksa.12245 38741370

[18] Kim J‐H , Chung J‐H , Lee D‐H , Lee Y‐S , Kim J‐R , Ryu KJ , et al. Arthroscopic suture anchor repair versus pullout suture repair in posterior root tear of the medial meniscus: a prospective comparison study. Arthroscopy. 2011;27(12):1644–1653. 10.1016/j.arthro.2011.06.033 21982389

[19] Kim JS , Lee MK , Choi MY , Kong DH , Ha JK , Kim JG , et al. Rehabilitation after repair of medial meniscus posterior root tears: a systematic review of the literature. Clin Orthop Surg. 2023;15(5):740. 10.4055/cios21231 PMC10551690 37811518

[20] Krych AJ , LaPrade MD , Hevesi M , Rhodes NG , Johnson AC , Camp CL , et al. Investigating the chronology of meniscus root tears: do medial meniscus posterior root tears cause extrusion or the other way around? Orthop J Sports Med. 2020;8(11):2325967120961368. 10.1177/2325967120961368 PMC7645763 33209944

[21] LaPrade CM , James EW , Cram TR , Feagin JA , Engebretsen L , LaPrade RF . Meniscal root tears: a classification system based on tear morphology. Am J Sports Med. 2015;43(2):363–369. 10.1177/0363546514559684 25451789

[22] Maheshwer B , Wong SE , Polce EM , Paul K , Forsythe B , Bush‐Joseph C , et al. Establishing the minimal clinically important difference and patient‐acceptable symptomatic state after arthroscopic meniscal repair and associated variables for achievement. Arthroscopy. 2021;37(12):3479–3486. 10.1016/j.arthro.2021.04.058 33964390

[23] Moews LD , Thamrongskulsiri N , Morgan JT , Vega TF , Perez Lloveras GO , Casanova F , et al. Surgical timing and outcomes in medial meniscus posterior root tear repair: a systematic review. Am J Sports Med. 2025;54:03635465261426366. 10.1177/03635465261426366 41923442

[24] Moon H‐S , Choi C‐H , Jung M , Chung K , Jung S‐H , Byun J , et al. Association between insufficient restoration of meniscal tension during surgical repair of medial meniscus root tear and surgical outcomes: clinical implication of curtain‐cliff sign. Am J Sports Med. 2025;53(1):163–171. 10.1177/03635465241293733 39548736

[25] Moon H‐S , Choi C‐H , Jung M , Lee D‐Y , Hong S‐P , Kim SH . Early surgical repair of medial meniscus posterior root tear minimizes the progression of meniscal extrusion: 2‐year follow‐up of clinical and radiographic parameters after arthroscopic transtibial pull‐out repair. Am J Sports Med. 2020;48(11):2692–2702. 10.1177/0363546520940715 32730732

[26] Moore M , Levitt S , Lin CC , Wolfe I , Alaia E , Meislin R , et al. Clinical outcomes following transtibial medial meniscal root repair are maintained at long‐term follow‐up. Knee Surg Sports Traumatol Arthrosc. 2024;32(11):2959–2966. 10.1002/ksa.12321 38923098

[27] Muller B , Yabroudi MA , Lynch A , Lai C‐L , Van Dijk CN , Fu FH , et al. Defining thresholds for the patient acceptable symptom state for the IKDC subjective knee form and KOOS for patients who underwent ACL reconstruction. Am J Sports Med. 2016;44(11):2820–2826. 10.1177/0363546516652888 27474383

[28] Ozkoc G , Circi E , Gonc U , Irgit K , Pourbagher A , Tandogan RN . Radial tears in the root of the posterior horn of the medial meniscus. Knee Surg Sports Traumatol Arthrosc. 2008;16(9):849–854. 10.1007/s00167-008-0569-z 18536902

[29] Papalia GF , Za P , Saccone L , Franceschetti E , Zampogna B , Vasta S , et al. Meniscal extrusion: risk factors and diagnostic tools to predict early osteoarthritis. Orthop Rev. 2023;15:74881. 10.52965/001c.74881 PMC10184885 37197670

[30] Takase R , Ohsawa T , Hashimoto S , Kurihara S , Yanagisawa S , Hagiwara K , et al. Insufficient restoration of meniscal extrusion by transtibial pullout repair for medial meniscus posterior root tears. Knee Surg Sports Traumatol Arthrosc. 2023;31(11):4895–4902. 10.1007/s00167-023-07528-3 37573532

